# An automated high throughput solution for DNA extraction and bisulfite-conversion from high volume liquid biopsy specimens: sample preparation for epigenetic analysis

**DOI:** 10.1186/s13104-019-4595-3

**Published:** 2019-08-30

**Authors:** Sebastian Rausch, Oliver Hasinger, Thomas König, Anne Schlegel, Gunter Weiss

**Affiliations:** Epigenomics AG, Berlin, Germany

**Keywords:** Liquid biopsy, Cell free DNA, cfDNA, High throughput DNA extraction, Bisulfite conversion, DNA methylation, Automation, High volume, Liquid handling

## Abstract

**Objective:**

DNA methylation analysis via real-time PCR or other analytical techniques requires purified bisulfite converted DNA. We report on an automated high throughput solution for DNA extraction, bisulfite-conversion, and purification of 96 samples with an input volume of up to 3.5 mL of plasma or urine, using reagents from the commercially available Epi BisKit.

**Results:**

Magnetic bead-based DNA extraction, bisulfite conversion at high temperature, and efficient DNA purification was conducted on a customized commercially available liquid-handling platform. A highly interlaced 4 × 24 sample protocol was implemented for DNA extraction, elution in a 96-well plate, efficient bisulfite-conversion and extensive purification. The resulting bisulfite-converted DNA was stored in a 96-well format, ready for PCR set-up or other down-stream applications. The automated method is a walk-away solution for processing 96 samples in 7 h 30 min. Performance of the method was validated by comparison with the standard manual method of the Epi BiSKit using technical and biological samples. Overall DNA yield was assessed with a standardized β-actin assay. The automated workflow demonstrated equivalent performance to the manual method for technical, plasma and urine samples. It may provide a new standard for effective high-throughput preparation of bisulfite-converted DNA from a variety of high volume liquid biopsy specimens.

## Introduction

The ever-increasing interest in epigenetic analysis paired with the convenience of liquid biopsy sampling naturally leads to questions on quality, efficiency, and standardization of sample preparation techniques [1]. Aberrant DNA methylation detected in liquid biopsies is a clinically useful biomarker for detection of cancer in the colon, rectum, liver or lung [2–4].

With the increase in potential clinical and research applications for the analysis of cell free floating DNA (cfDNA) in body fluid samples, new methods for collection and handling of plasma and urine have been developed. To date, key challenges remain in developing automated methods to process the large sample volumes required for cfDNA analysis as well as in standardized highly efficient bisulfite conversion methods for DNA methylation analysis. Key technology elements are efficient DNA extraction from high volume liquid biopsy samples, complete bisulfite conversion and extensive DNA purification to yield bisulfite-converted DNA of sufficient quality and quantity for downstream PCR applications [5].

Here, we report on an automated high throughput solution for preparation of purified bisulfite-converted DNA (bisDNA) from 96 samples in parallel with an input volume of up to 3.5 mL liquid biopsies (plasma or urine). For magnetic bead-based DNA extraction, bisulfite conversion at high temperature, and efficient DNA purification the commercially available reagents of the Epi BiSKit (RUO) were used [6]. The automated method was validated and compared to the manual method [6] by means of measuring the amount of bisDNA using an adapted real time PCR assay for β-Actin [7].

## Main text

### Materials and methods

#### Samples

Technical and biological samples were prepared to allow validation of the automated workflow and comparison to a manual method. Technical samples were cell-line derived DNA spiked into matrix. Biological samples were plasma pools and urine samples as examples for liquid biopsy specimens. Additional file 1: Table S1 provides information on the type and number of samples used.

Technical samples (A–G, HC1, LC1, HC2, LC2) were prepared by spiking cell line derived DNA into a matrix of 50 g/L bovine serum albumin (BSA) in 1× TE buffer. Samples A–G are essentially repeated preparations (lots) of the same type. High concentration (HC1, HC2) and low concentration (LC1, LC2) samples were technical controls typically used as processing controls for run validity assessment.

Biological samples comprised two types of liquid biopsy specimens—plasma and urine. For plasma, 90 individual > 7 mL pools were prepared from plasma of more than 100 subjects, and split into 3.5 mL aliquots. Urine from 14 healthy donors was used for the comparison study. All urine samples were treated with 50 mM EDTA for preservation and inhibition of DNase activity. Additionally, 1:4 dilutions of each sample were prepared in 1× Tris–EDTA buffer to broaden the total range of DNA concentrations at the lower end. The resulting 28 different urine pools with volume > 14 mL were split into four 3.5 mL aliquots, such that repeated measurement per sample and method was possible.

#### Instrument setup

For automated sample processing on a Tecan Freedom EVO 200 liquid handling system, the work flow as described in the Instructions of Use of the Epi BiSKit [6] was adapted. The instrument was specifically equipped with alternating 5 mL and 1 mL dilutors for the requirements of high-volume and low-volumes. Large liquid volumes were handled with 5 mL disposable tips, smaller volumes were pipetted using 1000 µL and 350 µL disposable tips. The robotic manipulator was configured with a centric gripper to cope with the high weight of filled 24-well plates. The DNA extraction of high-volume liquid biopsy samples was performed in 24-well plates (hitplate25, HJ Bioanalytik). The binding of DNA to the magnetic particles in the extraction step was performed in parallel on four heated shakers (BioShake D30-T elm, QInstruments) equipped with flat adapter plates. Magnetic beads were captured on a 24-well magnet plate (MagPlate24, Alpaqua). The reduction in volume after resuspension of magnetic beads enabled transfer to a 96-well deep well plate (Abgene, ThermoFisher) and bead capture on a MAGNUM FLX plate. The final eluted bisDNA was stored in 96-well PCR plates (Abgene, ThermoFisher).

#### Data and analysis

The output of the method was purified bisulfite-converted DNA, which was measured with real-time PCR assay specific for the bisulfite-converted sequence of β-Actin with amplicon length 129 bp described in detail previously [7]. Results were determined by means of cycle threshold (CT) analysis. Where multiple PCR wells per processed sample were run, the mean of CT values over PCR wells was recorded. Analysis was primarily descriptive and performed using standard libraries of software R [8].

### Results

#### Implementation of the automated method

Development of the automated method was based on replicating the manual procedure for the preparation of purified, sulfonated bisulfite-converted DNA from plasma outlined in the Instruction for Use of the Epi BiSKit [6] on the liquid handling robot. A schematic representation of the 87 major processing steps illustrating the interlaced character of the procedure for time efficient processing of 96 high volume samples in parallel is shown in Additional file 1: Figure S1.

#### Within-run and between-run variation

Initially, the automated workflow was evaluated with technical samples to assess consistency and reproducibility of results. For that purpose, a total of 256 technical samples were processed in three runs. Additional file 1: Table S2 summarizes the results. One HC1 sample was excluded from analysis due to a processing error. The mean Ct values for sample types HC1 and LC1 were very consistent over three runs, differing by 0.1 and 0.2, respectively. Low within-run variation was observed for both sample types. Even less variation between runs was observed at between-run standard deviations SD = 0.01 for sample HC1 and SD = 0.09 for sample LC1, demonstrating excellent reproducibility.

#### Comparison of the automated method to the manual method

**Fig. 1:**
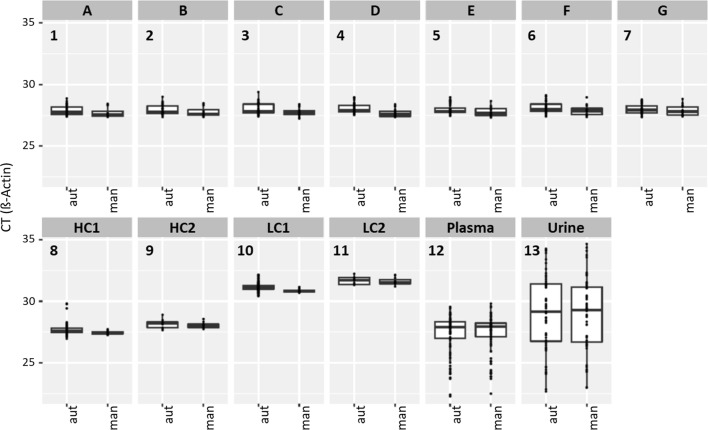
Comparative data for various sample types. Results shown for technical samples (**1**–**11**) and biological samples (**12**, **13**) for automated (left box plot) and manual (right box plot) method

Technical and biological samples were used to compare the automated method to the manual reference method (Additional file 1: Table S1). All technical samples were repeatedly processed in a number of automated and manual runs to compare DNA yield and reproducibility of the two methods. For demonstrating the equivalence of the two methods on biological specimens, one aliquot of each of 90 plasma pools was processed per method. Finally, for demonstrating the repeatability and equivalence of the two methods on a second type of liquid biopsy samples, two aliquots of each of 28 urine samples were processed per method. Figure 1 displays the full set of data for each of these sample types as paired box plots of CT results [automated method (left) and manual (right)]. As illustrated, the CT outcomes for the technical lots, the high and low controls and the liquid biopsy specimens were comparable between methods. As expected, for both the plasma and urine samples, a broad CT range was observed, representing the range of DNA concentrations typically observed for real world clinical specimens.

Additional file 1: Table S3 displays the number of replicates, the mean CT value and the corresponding standard deviation per method for the technical samples. For samples A–G, the results for both methods were very comparable at an overall difference in mean CT of < 0.2 and equivalent precision estimates of approximately SD = 0.4. Similarly, for the LC and HC samples, the results were very comparable with mean CT difference of < 0.2 for the four sample types. The observed precision was generally high despite the fact that two outlier results (among 75) had been observed on HC1 with the automated method.

**Fig. 2:**
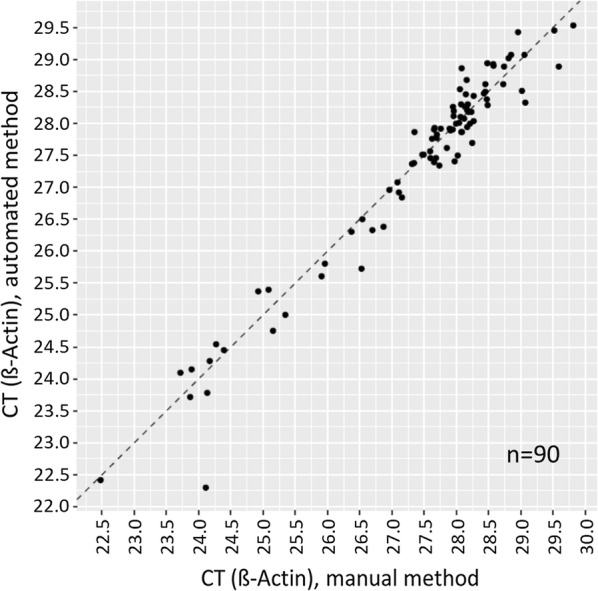
Paired results for 90 plasma samples processed with the automated and manual method. (dotted identity line; Pearson correlation r = 0.97)

For the plasma samples, correlation analysis of paired results for the two methods showed high overall agreement (Pearson correlation r = 0.97). The overall agreement for paired results is illustrated in Fig. 2, indicated by the narrow scattering of results near the identity line. An Altman-Bland analysis illustrated in Additional file 1: Figure S2 demonstrated a non-significant bias of 0.03 CT (SD = 0.35; p-value = 0.4).

**Fig. 3:**
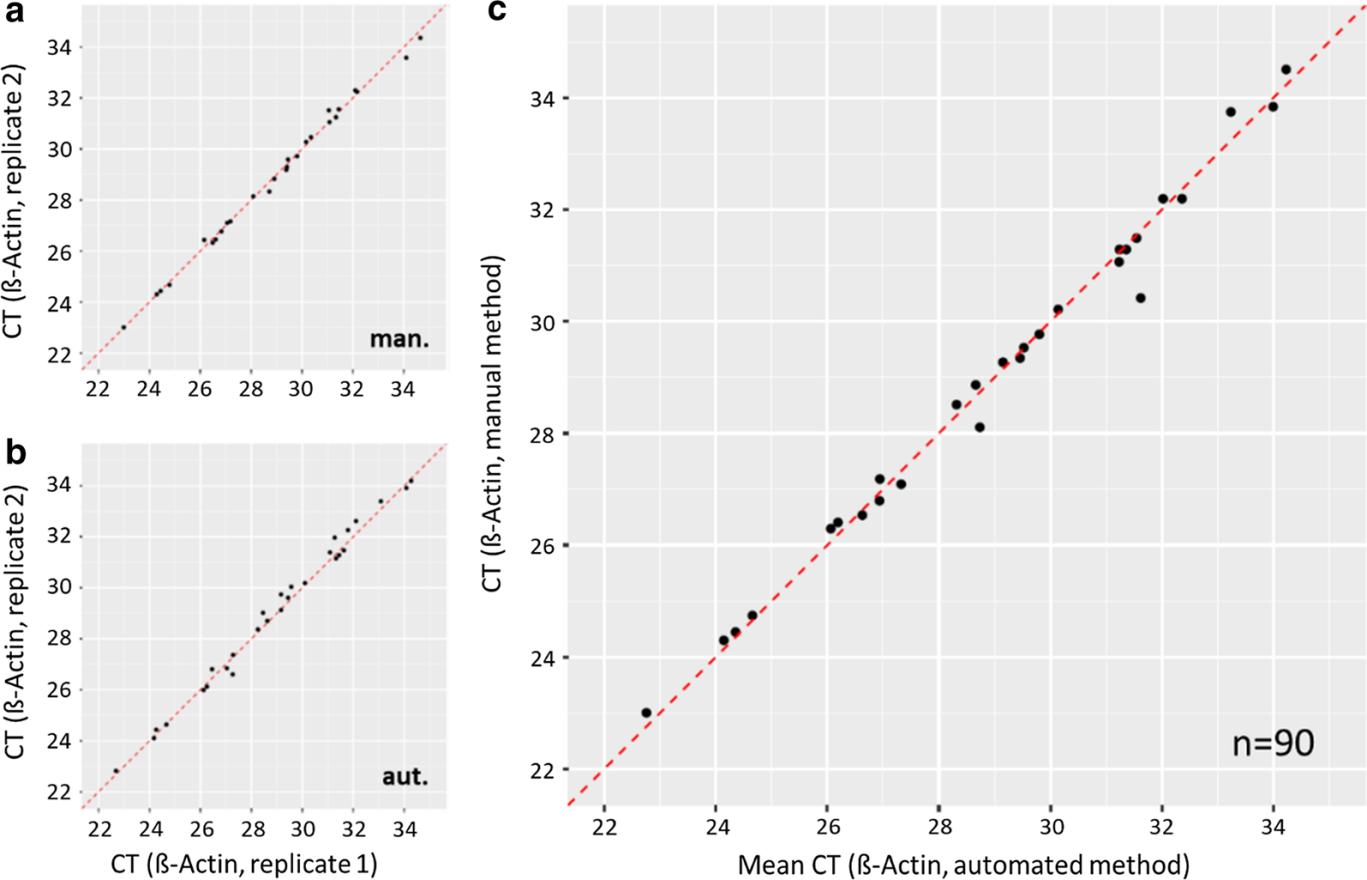
Paired results of 28 urine samples processed in duplicates with the manual or automated method. Left side: replicates within method (**a** manual; **b** automated). Right side: **c** comparison of results between methods

Urine samples were run in duplicate for each method, allowing within and between method comparisons. The correlation between replicates within either of the two methods was greater than 0.99 (Pearson correlation) illustrated in Fig. 3a, b. Intra-subject variability was low for both methods. Based on this excellent repeatability, the mean CT values of replicates were used for determining the correlation of results between the automated and the manual method for the urine data set. Figure 3c displays the paired results for both methods. Again, the observed correlation of results was excellent (Pearson correlation 0.99) and the Altman-Bland analysis provided a non-significant bias of − 0.01 CT (SD = 0.32; p-value = 0.9) (Additional file 1: Figure S3).

#### Reliability of the methods

Based on the total number of samples processed during the validation of the automated method, the success rate per method was used as a measure for reliability. For a total of 908 samples processed only 10 samples were excluded due to observed processing errors (pipetting issues induced by sporadic lumping events) resulting in a success rate of 98.9% (898/908) for the automated method. For the manual method a minimally smaller success rate of 98.1% was observed (298/314).

### Discussion

Large scale adoption of a method in the field is frequently supported by the availability of an automated procedure. Specifically challenging for high-throughput automation are samples of large volume (> 2 mL) which is a typical requirement for analysis of liquid biopsy specimens [5]. As a consequence, availability of automated procedures for extraction of DNA declines drastically for sample input volume above 1 mL and batch size beyond 24.

This automated method allows for input volumes of 3.5 mL and processes 96 samples in a single run within the limits of a typical work shift of 8 h. It uses reagents from the commercially available Epi BiSKit. It is a true walk-away solution and is the first method that fully integrates bisulfite-conversion and purification of DNA. The output, sulfonated bisulfite-converted DNA, is suitable for studying the phenomena of DNA methylation [9].

The Epi BiSKit made it specifically amenable to automation: all reagents are provided as liquids and the extraction method is based on magnetic particles. Still, adaptation of the work flow to automation presented a number of challenges: reaction volumes up to 10 mL in a 96-sample format during DNA extraction required handling of four 24 deep well plates in a highly interlaced process—being time efficient, but obeying time windows for incubation steps. As safeguard against impurities of biological samples leading to precipitates in the lysis reaction, treatment with Proteinase K was introduced to the automated procedure. Homogeneous mixture of reactions with largely diverse densities was achieved by applying pipette mixing steps before prolonged shaking incubations for all reactions, where shaking alone was not sufficient. The process of complete capture of magnetic particles was optimized while remaining within the time limits of the manual procedure. Bisulfite conversion takes place under harsh chemical conditions and requires a constant and uniform 80 °C temperature to be efficient while not damaging the DNA. To this end an adapter plate designed for the 96-well plates complemented with an optimized heating profile was implemented. Before the final elution of bisDNA, complete removal of residual ethanol by drying of magnetic particles at elevated temperature is essential for avoiding inhibition of subsequent PCR applications. As mentioned above the output of the method is purified, sulfonated bisulfite-converted DNA. Thus, some downstream applications might require an additional de-sulfonation step. Finally, a bar code reader was implemented for reagent and sample tracking. In combination, the automated method provides a real walk-away solution for processing 96 samples within approximately 7.5 h.

The method discussed here will be of interest to large research institutions and reference laboratories already performing large numbers of assays. Facilities having very high throughput capabilities will help to drive further development of liquid biopsy testing making use of epigenetic information like DNA methylation.

The automated workflow demonstrated equivalent performance on technical and biological specimens derived from plasma and urine over a broad range of DNA concentrations. Therefore, it may provide a new standard for effective high-throughput preparation of bisulfite-converted DNA from a variety of high-volume liquid biopsy specimens.

## Limitations

The method described has certain limitations. It is not fully flexible for the number of samples in a run. While a 48 sample version has been implemented, full flexibility would require substantial programming efforts. The method has not been used or validated for other body fluids like serum, saliva, pleural effusions or ascites. Finally, validation of the method has been performed in-house on a single instrument. However, consistent results are expected if an equivalent platform is properly installed and programmed prior to use.

## Supplementary information

**Additional file 1: Table S1.** Overview of samples and number of samples used. **Table S2.** Results from three automated runs with technical samples. **Table S3.** Comparative results from technical samples. **Figure S1.** Schematic representation of automated processing of 96 samples. **Figure S2.** Altman-Bland analysis of paired results for 90 plasma samples. **Figure S3.** Altman-Bland analysis of paired results for 28 urine samples.

## Data Availability

The datasets analysed during this study are available from the corresponding author upon reasonable request to the corresponding author.

## Abbreviations

cfDNA: cell free DNA
bisDNA: purified bisulfite-converted DNA
CT: cycle threshold
SD: standard deviation
