# Supplementary material for: An automated high throughput solution for DNA extraction and bisulfite-conversion from high volume liquid biopsy specimens: sample preparation for epigenetic analysis
Source: BMC Res Notes. 2019 Aug 30;12:551. doi: 10.1186/s13104-019-4595-3 (PMC6716871; doi:10.1186/s13104-019-4595-3)
Supplement: Supplementary file 1 — Additional file 1: Table S1. Overview of samples and number of samples used. Table S2. Results from three automated runs with technical samples. Table S3. Comparative results from technical samples. Figure S1. Schematic representation of automated processing of 96 samples. Figure S2. Altman-Bland analysis of paired results for 90 plasma samples. Figure S3. Altman-Bland analysis of paired results for 28 urine samples. [file 13104_2019_4595_MOESM1_ESM.docx]

**Additional File**

Table S1: Overview of samples and number of samples used.

Table S2: Results from three automated runs with technical samples.

Table S3: Comparative results from technical samples.

Figure S1: Schematic representation of automated processing of 96 samples.

Figure S2: Altman-Bland analysis of paired results for 90 plasma samples

Figure S3: Altman-Bland analysis of paired results for 28 urine samples

Table S1: Overview of samples and number of samples used.

| **Sample type** | **A – G** | **HC1** | **LC1** | **HC2** | **LC2** | **Plasma pools** | **Urine** | **total** |
| --- | --- | --- | --- | --- | --- | --- | --- | --- |
| **DNA conc. and type** | ~9ng/mL | 9ng/mL | 0.8ng/mL | 10ng/mL | 1ng/mL | NA | NA | NA |
| **Automated method** | 336 | 203 | 203 | 10 | 10 | 90 | 56 | 908 |
| **Manual method** | 112 | 17 | 17 | 11 | 11 | 90 | 56 | 314 |

Table S2: Results from three automated runs with technical samples.

|  | **Technical sample LC1** | | | **Technical sample HC1** | | |
| --- | --- | --- | --- | --- | --- | --- |
|  | Number of samples | ß-Actin mean CT | SD | Number of samples | ß-Actin mean CT | SD |
| Run 1 | 48 | 31.3 | 0.34 | 47 | 27.7 | 0.24 |
| Run 2 | 32 | 31.1 | 0.37 | 32 | 27.7 | 0.20 |
| Run 3 | 48 | 31.2 | 0.32 | 48 | 27.6 | 0.23 |
| Overall mean ß-Actin CT | | 31.2 |  |  | 27.7 |  |
| SD for between-run variation | | 0.09 |  |  | 0.01 |  |

Table S3: Comparative results from technical samples.

|  | **Automated Method** | | | **Manual Method** | | |
| --- | --- | --- | --- | --- | --- | --- |
| Technical Sample | Number of samples | ß-Actin mean CT | SD | Number of samples | ß-Actin mean CT | SD |
| **A** | 47 | 27.9 | 0.39 | 16 | 27.7 | 0.4 |
| **B** | 48 | 27.9 | 0.38 | 16 | 27.8 | 0.39 |
| **C** | 46 | 28 | 0.44 | 15 | 27.7 | 0.34 |
| **D** | 47 | 28 | 0.4 | 16 | 27.7 | 0.34 |
| **E** | 48 | 28 | 0.39 | 16 | 27.8 | 0.38 |
| **F** | 48 | 28.1 | 0.45 | 16 | 27.9 | 0.43 |
| **G** | 47 | 28 | 0.39 | 16 | 27.9 | 0.42 |
| **HC1** | 75 | 27.6 | 0.49 | 15 | 27.5 | 0.14 |
| **LC1** | 73 | 31 | 0.19 | 15 | 30.8 | 0.12 |
| **HC2** | 9 | 28.2 | 0.39 | 11 | 28.1 | 0.25 |
| **LC2** | 9 | 31.7 | 0.32 | 11 | 31.6 | 0.30 |


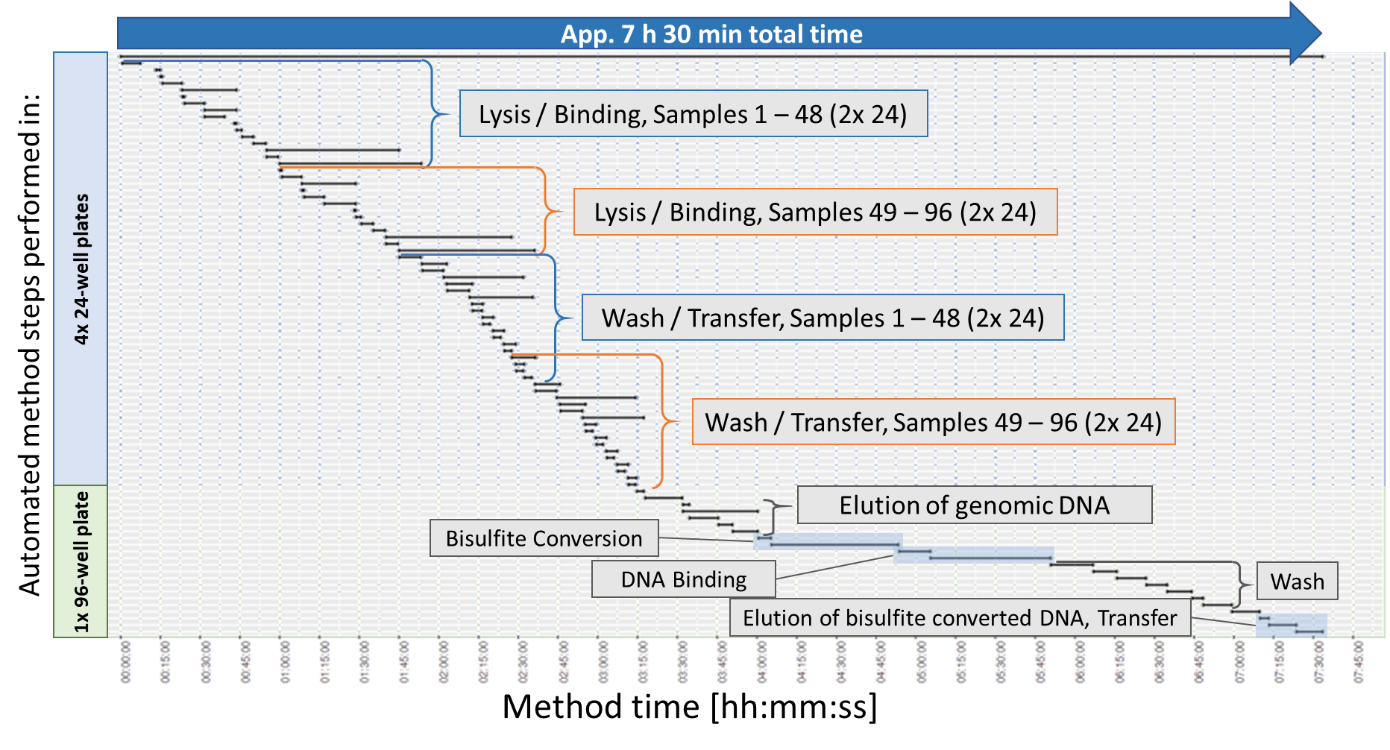


Figure S1: Schematic representation of automated processing of 96 samples. DNA extraction from high volumes is performed in 4x 24-well deep-well plates (blue grid background). Bisulfite conversion and purification are performed in a 96-well format (green grid background). The black bars indicate duration of individual steps. Overlapping of these time bars shows the highly interlaced sample processing in the 24-well plate format, avoiding instrument inactivity while maintaining required minimum incubation times


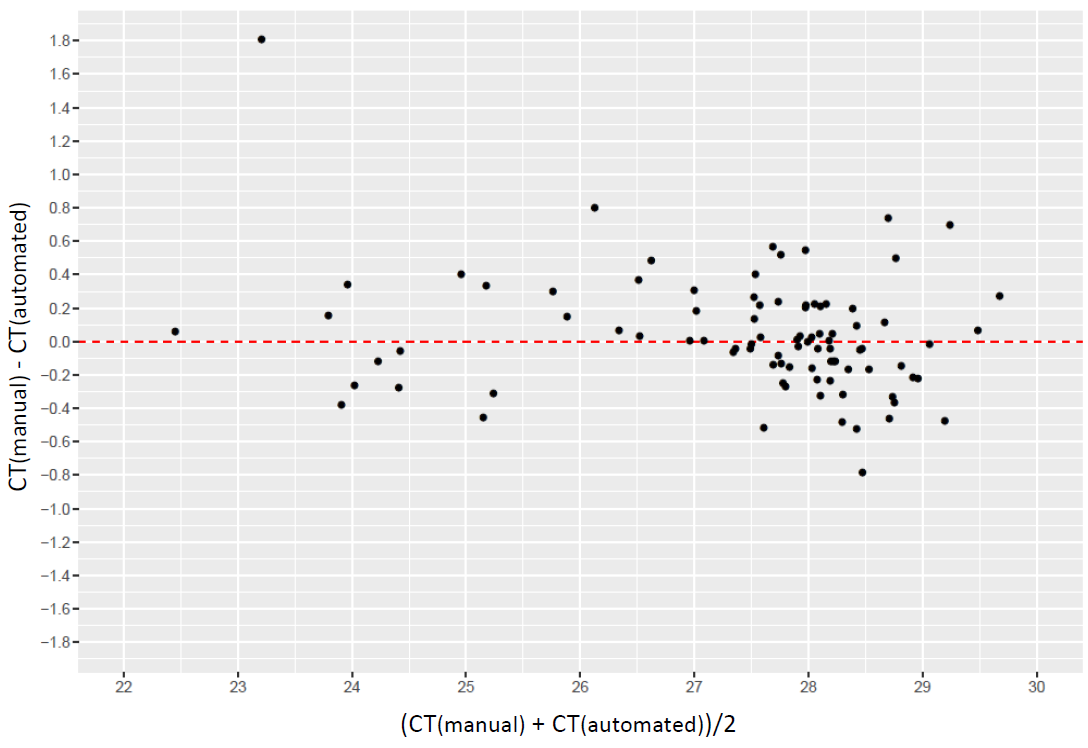


Figure S2: Altman-Bland analysis of paired results for 90 plasma samples processed with the automated and manual method (bias = 0.03 CT; SD = 0.35; p-value = 0.4).


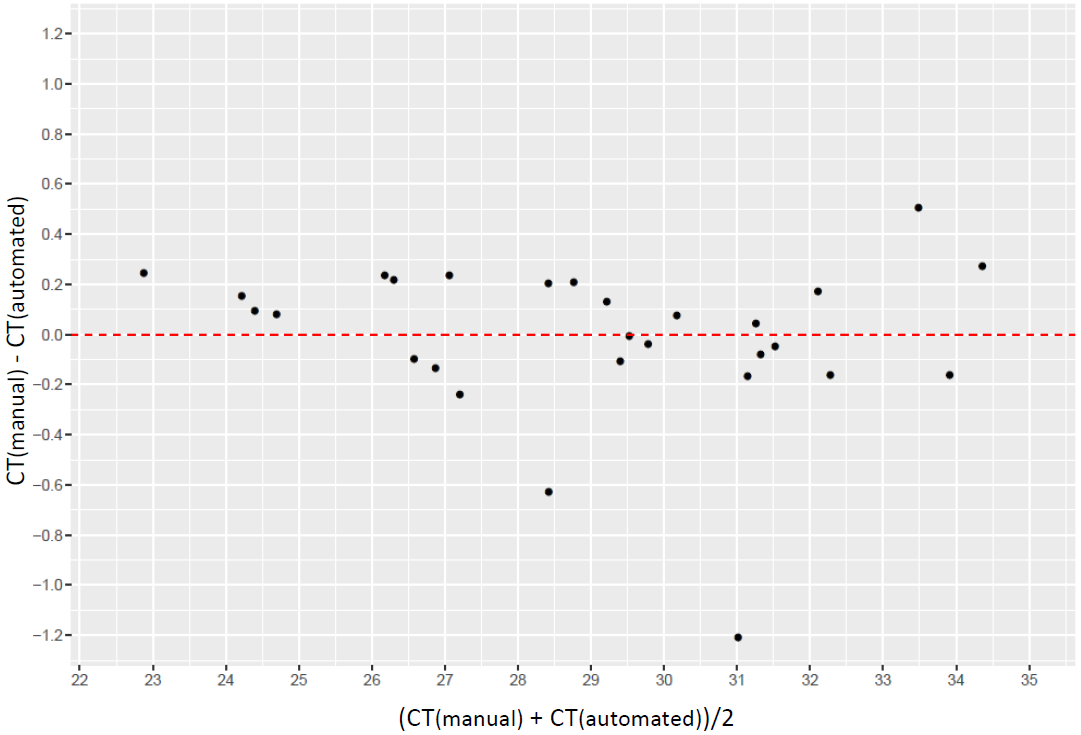


Figure S3: Altman-Bland analysis of paired results for 28 urine samples processed with the automated and manual method (bias = -0.01 CT; SD = 0.32; p-value = 0.9).
